# Neonatal lupus erythematosus as a rare trigger of gastrointestinal involvement in neonates

**DOI:** 10.1038/s41598-024-54091-z

**Published:** 2024-02-15

**Authors:** Changchang Fu, Wenqiang Sun, Hanghang Peng, Xueping Zhu

**Affiliations:** Department of Neonatology, Children’s Hospital of Soochow University, Suzhou, 215000 China

**Keywords:** Diseases, Medical research

## Abstract

Cutaneous and cardiac involvement in neonatal lupus erythematosus (NLE) has been extensively studied; however, gastrointestinal system involvement (GSI) remains unexplored. This study aimed to investigate the clinical features of GSI in patients with NLE with a particular focus on feeding intolerance (FI) and diarrhea. We conducted a retrospective analysis of the clinical data of patients diagnosed with NLE at the Children’s Hospital of Soochow University between 2011 and 2022. In this study, of 39 patients diagnosed with NLE, 27 presented with GSI. 9 patients who presented with FI or diarrhea as the primary manifestation were positive for anti-SSA antibody, and 5 were dual positive for anti-SSA and anti-SSB antibodies. Among the mothers of the NLE patients with GSI, 18 had systemic lupus erythematosus, 3 had Sjogren’s syndrome, 2 had mixed connective tissue disease, and one each had autoantibody abnormalities and photosensitivity symptoms; 4 mothers denied having any autoimmune disease. In this study, 69.23% of patients with NLE exhibited GSI, which was linked to hypocomplementemia and anti-SSA antibodies. Thus, clinicians should remain vigilant for NLE in neonates, particularly when accompanied with rash and other organ dysfunction and when the high-risk factors of FI and diarrhea have been excluded.

## Introduction

Neonatal lupus erythematosus (NLE), a rare, acquired autoimmune disease, is caused by the transplacental transmission of maternal antibodies to the fetus. The most common autoantibodies associated with NLE are anti-SSA and anti-SSB; however, a few patients may also test positive for anti-U1-RNP^1,2^. Transplacental antibodies typically lead to multiorgan damage in newborns and cause cutaneous, hematological, and cardiac manifestations, which may be accompanied by gastrointestinal and neurological involvement in some patients^2,3^. However, these clinical symptoms should be attributed to NLE only after excluding infectious diseases, hematological disorders, genetic metabolic disorders, and other non-NLE immune system diseases. Symptoms arising from noncardiac involvement in patients with NLE are typically transient and gradually improve after maternal antibody depletion^2,4^.

The common clinical manifestations of NLE patients include rash and arrhythmias^5,6^. Our previous studies have found that patients with NLE have predominant skin and hematological involvement^7^. However, it has been observed that gastrointestinal system involvement (GSI) often occurs along with other common manifestations. Gastrointestinal symptoms in patients with NLE are diverse, primarily comprising conjugated hyperbilirubinemia, elevated transaminases, feeding intolerance (FI), hepatomegaly, splenomegaly, and diarrhea, among others. These symptoms can be clinically confusing, often leading to misdiagnosis as gastrointestinal infection or other diseases and resulting in unnecessary tests and incorrect treatments.

## Methods

This study was designed as a retrospective cohort study of all patients with NLE who presented to the Children’s Hospital of Soochow University between January, 2011, and December, 2022.

In the present study, the criteria proposed by the American Rheumatology Association were used to establish NLE diagnosis^8^. Alternatively, unexplained damage to the liver and gallbladder or hematological abnormalities, along with the detection of serum anti-SSA or anti-SSB antibodies were considered diagnostic for NLE. All patients were assessed to exclude the non-NLE causes of gastrointestinal symptoms, including organic diseases, infectious diseases, and genetic metabolic disorders. Hematological routine tests, C-reactive protein (CRP), procalcitonin (PCT), stool routine analysis, and stool culture were performed to exclude gastrointestinal infections. Upon admission, all patients underwent upright abdominal X-ray and ultrasound examination. Moreover, patients with recurrent FI underwent gastrointestinal barium meal imaging to exclude organic gastrointestinal disorders. In some cases of recurrent FI/diarrhea, genetic metabolic disorders were excluded via blood and urine tandem mass spectrometry tests.

Data on the following parameters were collected from the patients’ medical records: demographic data, clinical characteristics, laboratory parameters, imaging findings, and history of maternal rheumatic disease. Laboratory tests included routine hematological and biochemical parameters, including aspartate aminotransferase (AST), alanine aminotransferase (ALT), and bilirubin; serological tests for immune rheumatoid factors; complement factors; cerebrospinal fluid analysis; electroencephalography; and electrocardiography. In addition, data were collected from various diagnostic imaging modalities, including X-ray examination (abdominal and pulmonary), magnetic resonance imaging (cranial), computed tomography (abdominal, pulmonary, and cranial), ultrasonography (abdominal and cranial), and echocardiography.

### Ethics statement

This study has already obtained an approval from the Ethics Committee of the Children’s Hospital of Soochow University (Ethics Approval Number: 2023CS024). The parents of all infants willingly agreed to participate in the study, and for clinical data disclosure, they provided written informed consent. All experiments involving the included human research participants were performed in accordance with the Declaration of Helsinki.

Statistical analysis was performed using the SPSS 26.0 software. Categorical data are presented as frequency (percentage). The pairwise comparisons of categorical variables were performed using Fisher’s exact test.

## Results

### Patients

Of the 39 patients with NLE included in the study, 18 were male and 21 were female. 27 (69.23%; 13 males and 14 females) patients with NLE had GSI, and their mean gestational age was 37 (range 35^+4^ to 37^+5^) weeks and mean birth weight was 2250 (range 1630–2760) g. Among the newborns with GSI, 11 (40.74%) were preterm, 9 (33.33%) were late preterm, and 3 (11.11%) were midterm. GSI manifestations were the initial presentation in 12 patients (30.77%), whereas 15 (38.46%) showed obvious symptoms only after diagnosis. The specific manifestations of GSI were conjugated hyperbilirubinemia (n = 18; 66.67%), elevated transaminases (n = 15; 55.56%), FI (n = 7; 25.93%), hepatomegaly (n = 7; 25.93%), splenomegaly (n = 5; 18.52%), diarrhea (n = 4; 14.81%), peritoneal effusion (n = 3; 11.11%), thrush (n = 2; 7.41%), and mouth ulcers (n = 1; 3.70%; Table 1).

**Table 1:** Demographic information and presenting symptoms of NLE patients with GSI (n = 27).

Characteristic	Classification	NLE without GSI (n = 12)	NLE with GSI (n = 27)	NLE (N = 39)
Gender	Male	6 (50.00%)	12 (44.44%)	18 (46.15%)
	Female	6 (50.00%)	15 (55.56%)	21 (53.85%)
Gestational age	≥ 37 weeks	5 (41.67%)	15 (55.56%)	20 (51.28%)
	< 37 weeks, ≥ 34 weeks	5 (41.67%)	9 (33.33%)	14 (35.90%)
	< 34 weeks	2 (16.67%)	3 (11.11%)	5 (12.82%)
Birth weight	< 1500 g	2 (16.67%)	4 (14.81%)	6 (15.38%)
	1500–2500 g	3 (25.00%)	12 (44.44%)	15 (38.46%)
	> 2500 g	7 (58.33%)	11 (40.74%)	18 (46.15%)
Gastrointestinal symptoms	Conjugated hyperbilirubinemia		18 (66.67%)	
	Elevated transaminases		15 (55.56%)	
	Feeding intolerance		7 (25.93%)	
	Hepatomegaly		7 (25.93%)	
	Splenomegaly		5 (18.52%)	
	Diarrhea		4 (14.81%)	
	Peritoneal effusion		3 (11.11%)	
	Thrush		2 (7.41%)	
	Mouth ulcers		1 (3.70%)	

*NLE* neonatal lupus erythematosus, *GSI* gastrointestinal involvement.

### Patients with NLE presenting with FI/diarrhea

9 patients presented with FI (n = 7) or diarrhea (n = 2; Table 2). The 7 patients with FI included two with diarrhea, 5 with rash, 5 with liver involvement, 4 with hematologic involvement, 3 with neurological involvement, and 2 with cardiac involvement. 3 of the 4 patients with diarrhea presented with rash, 3 had liver involvement, 2 had hematological involvement, and 1 had cardiac involvement. All 9 patients were positive for anti-SSA antibodies, whereas 5 were positive for both anti-SSA and anti-SSB antibodies. Of the 9 mothers, 4 had systemic lupus erythematosus (SLE), 2 had Sjogren’s syndrome, 1 had mixed connective tissue disease (MTCD), and 2 denied having a history of any autoimmune disease. None of the 9 patients had any pre-existing lesions of the digestive system or coinfection with serious illnesses and all were negative for enteropathogenic tests. Complete blood and urine analyses along with genetic testing for metabolic screening were performed only in one case with transient lysinuric protein intolerance. NLE was diagnosed based on the patient’s clinical features and maternal history of autoimmune disease in addition to autoantibody assay results.

**Table 2:** NLE patients with FI/diarrhea (n = 9).

No.	Gender	GA (weeks)	BW (grams)	Symptom	Organ involvement	Anti-SSA/SSB/U1-RNP	Maternal disease
1	F	37 + 4	2950	FI, rash, diarrhea	Gastrointestinal, neurological, cutaneous, hematologic, hepatobiliary	+/+/−	–
2	F	36 + 7	2439	FI, diarrhea	Gastrointestinal, hematologic	+/−/−	Sjogren's syndrome
3	F	37 + 5	2520	FI	Gastrointestinal, hematologic, cardiac	+/+/−	—
4	M	37 + 1	2500	FI, rash	Gastrointestinal, hepatobiliary, cutaneous, hematologic	+/+/−	SLE
5	M	35 + 2	1850	FI, rash	Gastrointestinal, cutaneous, cardiac, Hepatobiliary, hematologic, neurological	+/−/+	Sjogren's syndrome
6	F	36 + 3	2700	FI, rash	Gastrointestinal, cutaneous, hepatobiliary	+/−/−	SLE
7	M	38 + 5	2970	FI, rash	Gastrointestinal, cutaneous, hepatobiliary	+/−/+	SLE
8	F	29 + 6	1300	Diarrhea, rash	Gastrointestinal, cutaneous, cardiac hepatobiliary	+/+/−	MTCD
9	F	37 + 5	2550	Diarrhea, rash	Gastrointestinal, cutaneous, hematologic, hepatobiliary, neurological	+/+/−	SLE

*NLE* neonatal lupus erythematosus, *FI* feeding intolerance, *GA* gestational age, *BW* birth weight, *F* female, *M* male, *SLE* systemic lupus erythematosus, *MTCD* mixed connective tissue disease.

### Organ involvement in patients with NLE

With respect to multiple systems involved in the 39 patients with NLE, cutaneous involvement (79.49%) was the most common, followed by gastrointestinal (69.23%), hematological (64.10%), cardiac (53.85%), and neurological (25.64%) involvement, in that order. Among patients without gastrointestinal involvement (n = 12), the most common manifestations were cutaneous (66.67%), followed by cardiac (58.33%), hematological (58.33%), and neurological (25.00%) manifestations, in that order. Likewise, among patients with GSI (n = 27), cutaneous (85.19%), hematological (66.67%), cardiac (51.85%), and neurological (25.93%) involvement were the most common. In patients with GSI, the hematological manifestations included anemia (59.26%), hypocomplementemia (55.56%), neutropenia/agranulocytosis (37.04%), thrombocytopenia (25.93%), coagulation abnormalities (11.11%), and hypoproteinemia (14.81%; Table 3). Of the 18 patients with hyperbilirubinemia, 16 underwent phototherapy and 5 patients received low-dose steroid treatment for other systemic involvement. Among them, serum bilirubin levels returned to normal in 12 patients within 1–2 weeks. Moreover, serum bilirubin levels normalized in 16 patients before their discharge, whereas 2 exhibited a slight elevation that did not necessitate intervention.

**Table 3:** Organ involvement of NLE patients with gastrointestinal involvement (GSI).

Characteristic	Classification	NLE without GSI (n = 12)	NLE with GSI (n = 27)	NLE (n = 39)
Cutaneous (rash)	Total	8 (66.67%)	23 (85.19%)	31 (79.49%)
Gastrointestinal	Total	0 (0.00%)	27(100.00%)	27 (69.23%)
Hematologic	Total	7 (58.33%)	18 (66.67%)	25 (64.10%)
	Anemia	5 (41.67%)	16 (59.26%)	21 (53.85%)
	Hypocomplementemia	2 (16.67%)	15 (55.56%)	17 (43.59%)
	Neutropenia/deficiency	3 (25.00%)	10 (37.04%)	13 (33.33%)
	Thrombocytopenia	4 (33.33%)	7 (25.93%)	11 (28.21%)
	Coagulation abnormalities	2 (16.67%)	3 (11.11%)	5 (12.82%)
	Hypoproteinemia	1 (8.33%)	4 (14.81%)	5 (12.82%)
Cardiac	Total	7 (58.33%)	14 (51.85%)	21 (53.85%)
	Congenital heart block	2 (16.67%)	3 (11.11%)	5 (12.82%)
	1st degree AV block	0 (0.00%)	1 (3.70%)	1 (2.56%)
	2nd degree AV block	1 (8.33%)	0 (0.00%)	1 (2.56%)
	3rd degree AV block	1 (8.33%)	2 (7.41%)	3 (7.69%)
	Structural cardiac abnormalities	5 (41.67%)	11 (40.74%)	16 (41.03%)
	ASD/VSD	2 (16.67%)	3 (11.11%)	5 (12.82%)
	PDA	1 (8.33%)	4 (14.81%)	5 (12.82%)
	PFO	2 (16.67%)	4 (14.81%)	6 (15.38%)
Neurological	Total	3 (25.00%)	7 (25.93%)	10 (25.64%)
	Intracranial hemorrhage	2 (16.67%)	5 (18.52%)	7 (17.95%)
	Convulsions	2 (16.67%)	3 (11.11%)	5 (12.82%)
	Hydrocephalus	0 (0%)	4 (14.81%)	4 (10.26%)
	Extracerebral interval widening	0 (0%)	3 (11.11%)	3 (7.69%)
	Aseptic meningitis	0 (0%)	1 (3.70%)	1 (2.56%)

*NLE* neonatal lupus erythematosus, *GSI* gastrointestinal involvement, *AV* atrioventricular, *ASD* Abbreviations, *VSD* ventricular septal defect, *PDA* patent ductus arteriosus, *PFO* patent foramen ovale.

Some patients with GSI had cardiac involvement. 3 (11.11%) patients with GSI presented with cardiac conduction abnormalities: 1 with first-degree AV block and 2 with third-degree AV block. 11 (40.74%) patients with GSI had structural abnormalities, namely, atrial septal defect (ASD)/ventricular septal defect (VSD) (n = 3; 11.11%), patent ductus arteriosus (PDA) (n = 4; 14.81%), and patent foramen ovale (PFO) (n = 4; 14.81%). Meanwhile, 7 (25.93%) patients with GSI had neurological involvement, including intracranial hemorrhage (18.52%), convulsions (11.11%), hydrocephalus (14.81%), extracerebral interval widening (11.11%), and aseptic meningitis (3.70%). Furthermore, patients with GSI had a significantly higher incidence of hypocomplementemia than those without GSI (2/12 vs. 15/27; *P* = 0.037; Table 3).

### Epidemiology of antibodies and maternal history of autoimmune diseases in NLE patients with GSI

Among the 39 included patients with NLE, 30 (76.92%) tested positive for anti-SSA antibodies, 18 (46.15%) tested positive for anti-SSB antibodies, and 10 (25.64%) tested positive for U1-RNP. In addition, 14 patients (35.90%) were dual positive, i.e., had both anti-SSA and anti-SSB antibodies. Among the NLE patients with GSI, 25 (92.59%) were positive for anti-SSA antibodies, 13 (48.15%) were positive for anti-SSB antibodies, and 6 (22.22%) were positive for U1-RNP. Furthermore, 12 (44.44%) of these patients with GSI were positive for both anti-SSA and anti-SSB antibodies. Of note, patients with NLE and GSI had a significantly higher positive rate of anti-SSA antibodies than those without GSI (25/27 vs. 5/12, *P* = 0.001; Table 4).

**Table 4:** Epidemiology of antibodies and maternal diagnosis in NLE patients with GSI.

	NLE with GSI (n = 27)	NLE without GSI (n = 12)	NLE (n = 39)
Anti-SSA	25 (92.59%)	5 (41.67%)	30 (76.92%)
Anti-SSB	13 (48.15%)	5 (41.67%)	18 (46.15%)
U1-RNP	6 (22.22%)	4 (33.33%)	10 (25.64%)
SLE	18 (66.67%)	7 (58.33%)	23 (58.97%)
Sjogren's syndrome	3 (11.11%)	0 (0.00%)	3 (7.69%)
Photosensitivity symptoms	1 (3.70%)	2 (16.67%)	3 (7.69%)
MTCD	2 (7.41%)	0 (0.00%)	2 (5.13%)
Abnormal autoantibodies	1 (3.70%)	0 (0.00%)	1 (2.56%)
–	4 (14.81%)	3 (25.00%)	7 (17.95%)

*NLE* neonatal lupus erythematosus, *GSI* gastrointestinal involvement, *SLE* systemic lupus erythematosus, *MTCD* mixed connective tissue disease.

Among the 39 mothers of patients with NLE, 23 had SLE (58.97%), 3 had Sjogren’s syndrome (7.69%), 3 had photosensitivity symptoms (7.69%), 2 had MTCD (5.13%), 1 had abnormal autoantibodies (2.56%), and 7 (17.95%) denied having any history of autoimmune diseases. Among the mothers of those NLE patients with GSI, 18 (66.67%) had SLE, 3 (11.11%) had Sjogren’s syndrome, 2 (7.41%) had MTCD, 1 (3.70%) had abnormal autoantibodies, and 1 (3.70%) had photosensitivity symptoms; 4 (14.81%) of these mothers denied having any history of autoimmune diseases (Table 4).

### Follow-up

In general, follow-up data were obtained during outpatient visits or via telephone calls. A total of 28 cases were followed up. At the age of 2 months, 1 patient presented with a worsened generalized rash and mild liver function impairment. However, after steroid hormone treatment, the rash improved and completely resolved by the time the patient turned 5 months old. At the age of 9 months, all 28 infants showed normal blood indices and liver function, with skin rashes completely subsided. Moreover, 6 patients demonstrated different levels of growth retardation. At the age of 1 year, 23 infants were examined for autoantibodies and 22 tested negative.

## Discussion

NLE is a neonatal condition caused by the transplacental transmission of maternal antibodies, which can lead to multiorgan damage in the fetus/newborn. The main antibodies involved in NLE are anti-SSA and anti-SSB^5^. The incidence of NLE in offspring born to mothers with anti-SSA and anti-SSB antibodies has been reported as approximately 2%. Of these, < 5% of patients with NLE progress to SLE during adolescence or adulthood^9^. Earlier studies reported a higher risk of NLE development among females. However, subsequent studies revealed an equivalent risk among males and females^9,10^. In the present study, no significant sex difference was noted in patients with NLE.

Multiorgan involvement in NLE is characterized by the reversible impairment of the cutaneous, hematological, and gastrointestinal systems and partially irreversible blockade of cardiac electrical conduction. Among these manifestations, cutaneous and hematological manifestations are more common^5,11–13^. Clinical studies on gastrointestinal involvement in patients with NLE are relatively scarce. In the current study, organ involvement in the 39 patients with NLE, including 27 with concomitant GSI, was most commonly manifested as cutaneous and hematological involvement. The prevalence of cutaneous, hematological, and neurological involvement was higher in NLE patients with GSI than in those without GSI. Although the prevalence of cardiac involvement was lower in NLE patients with GSI than in those without, the difference was not statistically significant. Compared to patients with NLE but without GSI, a higher proportion of those with GSI had anemia, hypocomplementemia, neutropenia/deficiency, thrombocytopenia, and hypoalbuminemia, in terms of hematological manifestations. However, the differences were statistically significant only in the case of hypocomplementemia. Xu et al. reported that decreased C3 and CH50 levels are the independent risk factors of SLE combined with GSI^14^. Neurological involvement in NLE is rare, with cerebral edema being the most frequently reported manifestation. Few studies have also reported macrocephaly, stroke, and aseptic meningitis^6,15,16^. In the present study, neurological manifestations were intracranial hemorrhage, seizures, cerebral edema, widened extra-axial spaces, and aseptic meningitis. Of note, cerebral edema, widened extra-axial spaces, and aseptic meningitis were noted in patients with GSI; however, this association may have resulted from the small sample size.

Most studies have noted that the common symptoms of NLE are rash and cardiac arrhythmias^5,6^. However, in the current study, apart from rash and cardiac arrhythmias, additional gastrointestinal symptoms, including FI, diarrhea, and ascites, were observed in some patients. This co-occurrence may be attributed to mesenteric vasculitis caused by lupus antibodies and hepatic dysfunction^17,18^. SLE affects the entire gastrointestinal system, including the stomach, intestines, liver, and pancreas, all of which are directly or indirectly associated with SLE^18^. GSI in SLE can occur early or late in the course of the disease. In their study, Bader-Meuner et al.^19^ reported that 17% of adolescent patients with SLE initially presented with gastrointestinal symptoms. Fawzy et al.^20^ found that 42.5% of pediatric patients with SLE had concurrent GSI. In the present study, 27 (69.23%) patients with NLE had GSI, with 12 (30.77%) having gastrointestinal symptoms at initial presentation. 15 (38.46%) of these patients developed gastrointestinal symptoms after diagnosis, which is a rate significantly higher than that reported in previous studies. This high rate may be related to the immature development of the neonatal gastrointestinal system, abundant intestinal mucosal vasculature, and weaker mucosal barrier, making neonates more susceptible to the effects of lupus antibodies. Oral ulcers caused by SLE are relatively rare and typically present as painless ulcers that are often located on the hard palate^21^. In the present study, only one patient had a painless oral ulcer on the hard palate, which resolved spontaneously after approximately 2 weeks.

To date, no specific antibodies have been directly associated with GSI in SLE^22^. Sönmez et al.^23^ revealed that ENA may be associated with GSI in adolescent patients with SLE. In the present study, the positivity rate of anti-SSA antibodies in patients with NLE and GSI was significantly greater than that in those without GSI, suggesting a potential association between anti-SSA and GSI in these patients. Most mothers of patients with NLE have autoimmune diseases, including SLE, although a small portion of mothers may not have any history of autoimmune diseases^24,25^. In the current study, the most common autoimmune disorder among the mothers of the included patients was SLE, followed by Sjogren’s syndrome, MTCD, photosensitivity symptoms, and autoantibody abnormalities. Seven mothers denied having any autoimmune diseases. Therefore, when newborns present with multiple organ involvement resembling NLE, they should be carefully evaluated, particularly for NLE, even if their mothers have no relevant history of autoimmune diseases or are negative for autoantibodies such as anti-SSA/SSB antibodies. Of note, both Sjogren’s syndrome and MTCD occurred in the mothers of patients with NLE and GSI, suggesting a potential association between these conditions and GSI in NLE.

The present study assessed the clinical characteristics, antibody epidemiology, and maternal history of autoimmune diseases in patients with NLE and GSI and provided relevant insights that can guide future research. However, this study has some limitations. First, this was a single-center study with a relatively small sample size, which may have introduced bias in the research conclusions. Second, neonatal conjugated hyperbilirubinemia is a common occurrence, and it remains uncertain whether all cases can be attributed to NLE. This uncertainty may have impacted the validity of the results. Third, this study was retrospective and all children did not undergo comprehensive or dynamic monitoring with abdominal and neuroimaging examinations, possibly introducing bias in the results. Therefore, in the future, multicenter, collaborative, prospective studies are warranted for more reliable data that can provide appropriate guidance for the clinical diagnosis and treatment of NLE.

## Conclusions

In summary, 69.23% of patients with NLE in this study had GSI, and GSI was associated with the presence of hypocomplementemia and anti-SSA antibodies. Some patients had gastrointestinal symptoms, including FI and diarrhea, at initial presentation. Therefore, clinicians should be alert to the possibility of NLE when patients present with gastrointestinal-related phenotypes during the neonatal and infancy period, particularly when they are accompanied by a rash or dysfunction of other organ systems. Moreover, other causes of these symptoms should be excluded via detailed inquiry regarding the maternal history of autoimmune diseases and timely autoantibody testing, particularly anti-SSA/SSB antibodies.

## Data Availability

The original contributions presented in the study are included in the article/supplementary material, further inquiries can be directed to the corresponding authors.
